# Multifunctional Application of Biopolymers and Biomaterials

**DOI:** 10.3390/ijms241210372

**Published:** 2023-06-20

**Authors:** Swarup Roy, Valentina Siracusa

**Affiliations:** 1 Department of Food Technology and Nutrition, School of Agriculture, Lovely Professional University, Phagwara 144411, India; 2 Department of Chemical Sciences, University of Catania, Viale Andrea Doria 6, 95125 Catania, Italy; vsiracus@dmfci.unict.it

Biopolymers and biomaterials are two interconnected key topics, which have recently drawn significant attention from researchers across all fields, owing to the emerging potential in multifunctional use. Biopolymers are commonly used to fabricate many biomaterials and, moreover, the combination of biomaterials and biopolymers can produce a new generation of materials for versatile applications. Biopolymers are a topic of interest nowadays, not only due to their potential applications in food, pharmaceuticals, textiles, medicals and other sectors but also to address the challenges of upward environmental pollution [1]. The unorganized use of readily available and cost-effective synthetic plastic has already caused severe damage to the environment and, now, these plastics are becoming a serious threat to all living beings on the planet [2,3]. The tremendous use of synthetic plastic in all daily used items is becoming a serious threat to us and, thus, there is a need for an instant alternative, and, in this context, bio-based sustainable and degradable polymers have high potential to replace the petrochemical-derived synthetic polymers. There are various biopolymers, such as protein, polysaccharides or their combinations, that are commonly used to develop bioplastics. Biopolymer-based polymers have comparable properties like their synthetic counterparts [4,5]. The addition of functional materials, such as nanomaterials, essential oils, phytochemicals, bioactive components, etc., helps in further improvements in both physical and functional properties of the biopolymer-based materials [6,7,8]. Recent research has shown that biopolymer-based functional packaging (active and intelligent packaging) film and coatings have good potential to improve the life span of packed food items [9,10]. Moreover, biopolymers have also been used as hydrogel and dressing materials to treat wounds in the biomedical sector [11,12]. Even biomaterials are commonly used to fabricate medical devices [13].

Biomaterials include, but are not limited to, synthetic or natural polymers, ceramics and composites, which can interact with biological matter. Recently, biomaterials have been increasingly used and, day by day, they are substituting the conventional polymeric materials in agriculture, textile, medical, food, cosmetics and pharmaceutical sectors [14,15,16,17]. The medical sector is the most used market for biomaterials [18]. Nowadays, in the pharmaceutical and textile sectors, biomaterials are also readily available. Many biopolymer-based biomaterials are used as micro- and nanofibers in the textile sector [19]. Biomaterials are frequently used in drug delivery systems in the pharmaceutical industry. Moreover, there is growing public concern for the environment and health; indeed, many folds replicate the use of biomaterials in various sectors. Even though biomaterials and biopolymers are emerging very rapidly, as already discussed, more research is still needed for further development in this field. Nevertheless, the complete conversion of conventional materials to biomaterials is expected to take more time.

Biomaterials and biopolymers are promising for making sustainable materials, but there are many concerns, which need more attention before further progress. The cost of these materials is higher than the traditionally used ones, which restricts the use in many sectors. More in-depth knowledge about biopolymers and biomaterials is required to produce futuristic materials. Furthermore, there is a need for improvements in the physical properties of the biomaterials to meet the requirement in industrial-level applications. The ongoing and future research on this topic is anticipated to help in developing more sustainable materials.
